# Antibiotic Susceptibility Profile and Tetracycline Resistance Genes Detection in *Salmonella* spp. Strains Isolated from Animals and Food

**DOI:** 10.3390/antibiotics10070809

**Published:** 2021-07-02

**Authors:** Valeria Gargano, Sonia Sciortino, Delia Gambino, Antonella Costa, Vincenzo Agozzino, Stefano Reale, Rosa Alduina, Domenico Vicari

**Affiliations:** 1 Istituto Zooprofilattico Sperimentale della Sicilia, 90129 Palermo, Italy; valeria.gargano@izssicilia.it (V.G.); antonella.costa@izssicilia.it (A.C.); v.agozzino@libero.it (V.A.); stefano.reale@izssicilia.it (S.R.); domenico.vicari@izssicilia.it (D.V.); 2 Department of Biological, Chemical and Pharmaceutical Sciences and Technologies (STEBICEF), University of Palermo, 90028 Palermo, Italy; valeria.alduina@unipa.it

**Keywords:** *Salmonella*, antibiotic resistance, ARGs, *tet* genes, animals, food

## Abstract

*Salmonella* spp. is among the leading causes of foodborne infections in humans and a large number of animals. *Salmonella* spp. is a pathogen involved in the dissemination of antimicrobial resistance because it can accumulate antibiotic resistance genes (ARGs). In this study, the antibiotic resistance profile to 15 antibiotics, belonging to six different classes, of 60 strains of *Salmonella* spp. collected from pets, farm animals, wildlife, and food in Sicily (Italy) was investigated by the Kirby-Bauer method. Given that almost 33.3% of the *Salmonella* spp. strains were resistant to tetracycline, Real-Time PCR analysis was applied on all the 60 strains to detect the presence of eight selected *tet* resistance genes. Besides, the presence of the *int1* gene, related to the horizontal gene transfer among bacteria, was also investigated in all the strains by Real-Time PCR analysis. Our data showed that 56% of the isolated strains harbored one or more *tet* resistance genes and that these strains were most frequently isolated from animals living in close contact with humans. Concerning *int1*, 17 strains (28.3%) harbored this genetic element and eight of these simultaneously contained *tet* genes. The results of this study highlight the importance of using a molecular approach to detect resistance genetic determinants, whose spread can increase the diffusion of multidrug-resistant strains. Besides, the study of zoonotic bacteria such as *Salmonella* spp. which significantly contribute to ARGs dissemination should always follow a One Health approach that considers the health of humans, animals, and the environment to be closely related.

## 1. Introduction

The genus *Salmonella*, represented by facultative anaerobe bacilli with flagella, includes 2579 different serotypes divided into two species, *S. enterica* and *S. bongori* [1]. Bacteria belonging to the genus *Salmonella* are etiological agents with *S.* Typhimurium and *S.* Enteritidis, which cause gastrointestinal infections [2]. Non-typhoidal *Salmonella* infections can occur in pets, including domestic reptiles (iguanas and aquatic turtles), farm animals (chickens, pigs, cattle, rodents, dogs, cats, chicks), wildlife, and humans; in the latter, they are responsible for more than 50% of total gastrointestinal infections and are one of the most frequent causes of foodborne outbreaks in the industrialized world. Animals are often asymptomatic or may have subclinical forms of infection, and can contribute to environmental contamination through the faecal release of *Salmonella* [3]. This infectious disease can reach humans from production animals through the food chain, by direct contact with animals or by exposure to water or crops contaminated with manure [4]. Household, nonconventional pets (e.g., hedgehogs) and wild animals can also have a role in human salmonellosis [5]. Besides, reptiles, frequently found as common pets, are considered a reservoir of *Salmonella* spp. and can be a source of non-typhoidal human salmonellosis [6]. The annual report on zoonoses and zoonotic agents edited by the European Food Safety Authority (EFSA) and the European Centre for Disease Prevention and Control (ECDC) provides an overview of the monitoring activities carried out in 36 European countries (28 EU Member States and 8 non-members). To date, *Salmonella* spp. infections represent the second largest zoonosis in UE. Additionally, the World Health Organization (WHO) recommends national and regional laboratories to conduct foodborne surveillance for this pathogen. The WHO is also promoting integrated antimicrobial resistance surveillance of pathogens in the food chain by collecting samples from humans, food, and animals and analyzing data across all sectors. Considering the pathogenic action of *Salmonella* and the possible role of animals as sources of food contamination and human infection, several European countries have implemented surveillance systems (Enter-Vet in Italy) to collect data on *Salmonella* spp. isolated from samples of veterinary origin.

Gastrointestinal infections caused by *Salmonella* spp. have a self-limiting course and are not treated with antibiotics in healthy subjects. However, in children, the elderly, and immunocompromised individuals, the use of these drugs is necessary to prevent serious systemic illness [7]. Tetracyclines are broad-spectrum antimicrobial drugs, exhibiting activity against a wide range of both Gram-positive and Gram-negative bacteria, and are currently used for therapy and prophylaxis for human infections and the prevention and control of bacterial infections in veterinary medicine [8]. The increasing incidence of resistance to tetracyclines in *Salmonella* spp. of human and animal origins has been reported worldwide [9]. The overuse of antibiotics for therapy and prophylaxis of human and food-producing animal infections has contributed to *Salmonella* spp. being resistant to many classes of antibiotics, including those of first choice for treating *Salmonella* infection in humans [10]. The misuse in veterinary medicine of tetracycline to treat infections in cattle, poultry, sheep, and swine, or even to promote the growth of some animal species, has contributed to the increase in both human and animal resistant isolates, and consequently to the greatly increased probability of clinical failure [11].

Therefore, antimicrobial resistance (AMR) is widespread in *Salmonella* spp. isolates, both of human origin and other sources [12]. AMR is increasing to dangerous levels in all parts of the world and is now a major risk to global health and food security [13,14,15,16]. AMR occurs naturally, but the overuse of antibiotics in humans and animals is accelerating the process [15,17]. Bacteria can become resistant after spontaneous changes or mutations in their genetic material [9] or through DNA acquisition from other bacteria, such as integrons (*int*) and plasmids that might play a key role in the spread of antibiotic resistance genes (ARGs) [18]. When a plasmid harboring ARGs is transferred into other bacteria, antibiotic resistance can spread easily and quickly among strains [19]. Besides, integrons, which are mobile genetic elements containing ARG cassettes, can integrate into chromosomes or plasmids by site-specific recombination [20,21].

In Italy, *Salmonella* spp. strains showed higher resistance profiles than the European average, with sulfamethoxazole being ineffective in 44.9% of cases, followed by tetracycline (40.4%) and ampicillin (37.4%) [22]. Moreover, the latest EFSA report showed alarming values of resistance to the critically important antimicrobial (CIA), ciprofloxacin and cefotaxime and/or ceftazidime of 18.9% and 23.5–31.6%, respectively [23].

Resistance to tetracycline can occur by several mechanisms: antibiotic efflux or modification, protection of the binding site or modification of 16S rRNA at the tetracycline-binding site [24].

So far, several *tet* genes that contribute to tetracycline resistance in *Salmonella* spp. have been described. The most frequent types of *tet* genes belong to classes A, B, C, D and G. Many of these genes can be localized within mobile portions of the *Salmonella* genome, such as transposons or plasmids, and this allows them to be easily disseminated in the environment and transferred to other bacteria [9,25].

According to WHO, antibiotic-resistant bacteria in Europe kill about 33 thousand people each year [26]. To protect human health, the only way forward is the proper use of antibiotics and a timely monitoring of the spread of antibiotic resistance and the genes that contribute to determining it. Bacteria belonging to the genus *Salmonella* spp. can represent tangible reservoirs of ARG genes and contribute greatly to the selection of multidrug-resistant (MDR) bacteria [27]. The AMR is now recognized as a zoonosis, and represents a problem not only in hospitals; indeed AMR strains have been isolated in confined environments where the selective pressure exerted by the use of antibiotics is low [28,29,30]. Therefore, this study aims to contribute to the analysis of antibiotic resistance profiles of *Salmonella* spp. strains isolated from animals and food of animal origin, and to investigate the spread of tetracycline resistance genes. In fact, in an insular territory such as Sicily, where livestock breeding is mainly of extensive/breeding type, the transmission of zoonotic pathogens can occur between farm animals, wild animals, pets and humans and can be monitored. For these reasons, following a “One Health” approach, we collected data on strains isolated from various contexts.

## 2. Results

### 2.1. Collected Salmonella Strains

A total of 60 strains of *Salmonella* spp. from animals (livestock, pet, zoo animals, wild) and food of animal origin were collected. The origin and serotypes of *Salmonella* spp. isolated are shown in Table 1.

### 2.2. Antimicrobial Susceptibility

All *Salmonella* isolates were susceptible to chloramphenicol, ciprofloxacin, cefotaxime, enrofloxacin, levofloxacin and ceftriaxone. None of the isolates showed resistance to clinically important antimicrobial agents, such as ceftriaxone, ciprofloxacin, amoxicillin-clavulanic acid and imipenem. The results of antibiograms performed by the Kirby-Bauer method are reported in the Supplementary Table S1.

**Figure 1:**
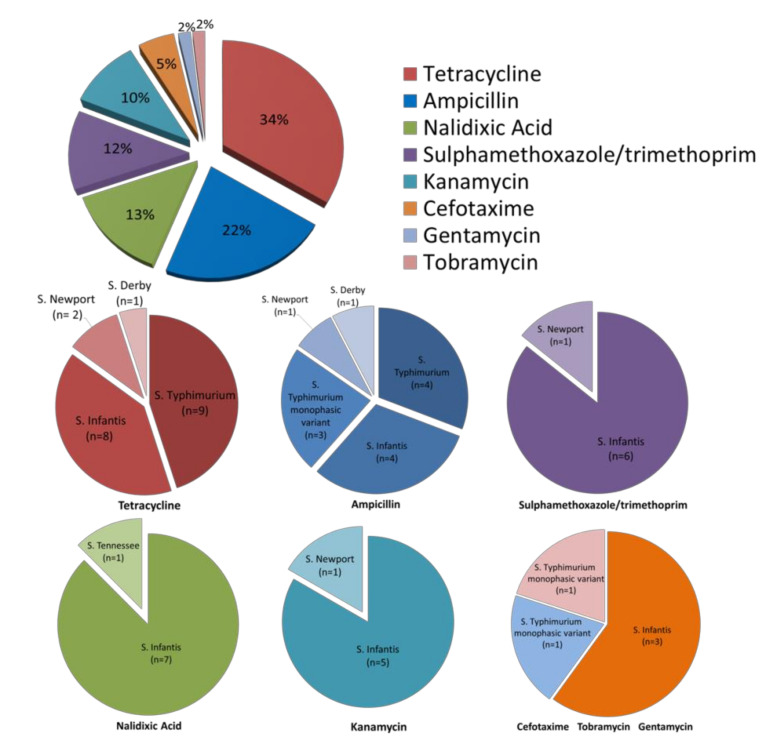
Phenotypic resistance profile of 60 *Salmonella* isolates.

33.3% (20/60) of the *Salmonella* isolates were resistant to tetracycline, with *S.* Typhimurium representing the majority of these isolates (9 isolates), and 21.6% (13/60) were resistant to ampicillin with *S.* Typhimurium (7 isolates) representing the most common serovar, as showed in Figure 1.

The first pie chart shows the percentage of phenotypic resistances of the 60 strains analyzed. The detail of the antibiotic-resistant serovars is shown in the pie charts below.

The details of the antibiogram results for each strain are shown in Table 2.

**Figure 2:**
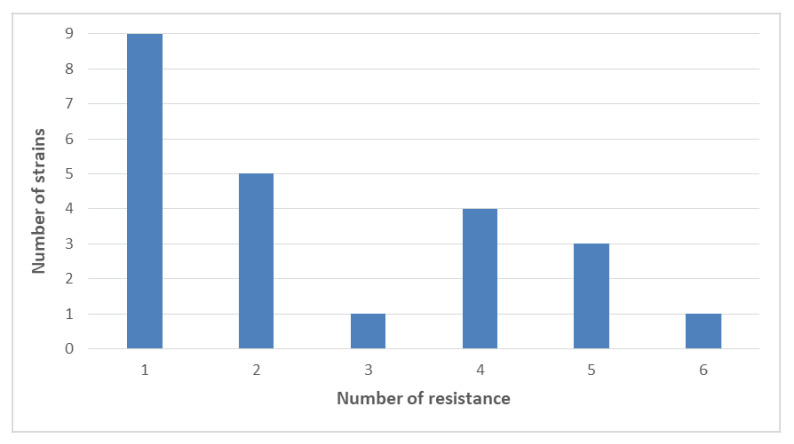
Number of resistant strains detected.

Multidrug resistance (MDR), defined as resistance to three or more tested classes of antibiotics, was observed in nine strains of *Salmonella* spp., with all these isolates resistant to tetracycline (Figure 2).

### 2.3. Detection of Tetracycline Resistance Genes and Class-1 Integron

Considering the percentage of tetracycline-resistant strains and the possibility of the spreading of ARGs by horizontal gene transfer, Real Time PCRs were conducted on the 60 *Salmonella* spp. strains to determine the presence of genetic determinants of tetracycline resistance and class 1 integron. This analysis showed that 41.7% (25/60) of the analyzed strains harbored one or more *tet* resistance genes: 10 strains were from pets, 5 strains from livestock, 5 strains from zoo animals, 4 strains from food, while only 1 strain was isolated from a wild animal.

Among the strains analyzed, *tet* (A) was the most prevalent gene, present in 56% (14/25) of the strains harboring at least one *tet* gene, and *tet* (A) gene was found alone in 9/14 strains, while it was in association with other *tet* genes in the remaining five strains.

The *tet* (G) was the second most represented gene, found alone or in association with other *tet* genes in 5/25 of the strains; while the *tet* (C) and *tet* (D) genes were found at the same frequency (4/25) among the analyzed strains; finally, *tet* (B) gene was found in 3/25 strains. The *tet* (E) gene was the least frequently detected, in fact it was harbored only by 2/25 strains. Concerning *int1* gene, it was found in 17/60 strains, 8 of which harbored at least one *tet* gene. Moreover, among the 25 strains that possessed at least one *tet* gene, only 11 were phenotipically resistant to tetracycline (Table 3) and nine tetracycline resistant strains did not contain any of the analyzed genes.

## 3. Discussion

This study demonstrates a larger diffusion of tetracycline resistance genes with respect to the tetracycline-resistance profile of 60 *Salmonella* spp. strains. The strains analyzed belonged to 44 isolated from animals (livestock, pets, zoo or wildlife) and 16 from food.

*S. enterica* was the most prevalent isolated species; in fact, only one *Testudo hermanni* harbored *S. bongori*. Among *S. enterica* strains, the prevalent serovars were Typhimurium (23.3%), including the monophasic variant, and Infantis (13.3%), as shown in Figure 1. Indeed, Typhimurium and Infantis serotypes are the most prevalent species found in humans and animals, and are considered “zoonotic salmonellae” [22]. Among the 60 strains, 23 (38.3%) showed resistance to at least one antibiotic among the 15 tested; in particular, the antibiotics for which a higher percentage of resistance was found were tetracycline (33.3%) and ampicillin (21.6%), widely used in human and veterinary medicine. In contrast, low rates of resistance were observed toward gentamicin and tobramycin (1.6%), cefotaxime (5%), kanamycin (10%), sulfamethoxazole/trimethoprim (11.6%), and nalidixic acid (13.3%).

Most of the strains that showed phenotypic resistance to tetracycline belonged to the serovars monitored by EFSA for antibiotic resistance [12]. According to the comprehensive antibiotic resistance database (https://card.mcmaster.ca, accessed on date 10 February 2021), a bioinformatic database of resistance genes, we have chosen eight of the most studied genes associated with tetracycline resistance, six of which encode for proteins involved in the regulation of efflux pumps, with two for proteins of ribosomal protection. By comparing results obtained using Real Time PCR to detect *tet* resistance genes with the results of tetracycline-resistance profile on 60 strains of *Salmonella* spp., we found that 41.6% (25/60) of the strains harbored one or more tetracycline resistance gene confirming what has been well documented by other studies [31,32]; conversely, the Kirby Bauer test evidenced that 33.3% of strains were resistant. Indeed, the presence of *tet* genes was revealed in 14 phenotypically susceptible strains. This result could be explained by the fact that not only efflux pumps contribute to tetracycline resistance but also other mechanisms, as suggested in other reports [33]. Moreover, we can surmise that the *tet* genes (*tets* (*A*) and (*B*)) detected in 14 tetracycline-sensitive strains are not expressed in the experimental conditions used in this study. Furthermore, the Kirby Bauer test showed the presence of nine MDR strains: one *S.* Typhimurium monophasic variant from pets, and one *S.* Infantis from food resistant to three classes; six *S.* Infantis and one *S.* Newport, all from food resistant to four classes; one *S.* Infantis from zoo animals resistant to five classes (Figure 2).

All *tet* genes detected in this study (*tet* (A), *tet* (B), *tet* (C), *tet* (D), *tet* (E), *tet* (G)) are implicated in the mechanism of tetracycline extrusion from the bacterial cell, that represents a common strategy of tetracycline resistance. We did not detect *tet* (O) and *tet (W)*, that were recently found to be diffused in Gram-negative bacteria isolated from water samples of various origin [34]. The most frequently detected gene was *tet* (A), harbored by 14/25 (56%) strains; this gene encodes a subunit of the tetracycline efflux pump found in many species of Gram-negative bacteria [35]. Thirteen of the strains that possessed *tet* (A), eight of which also showed phenotypic resistance to tetracyclines, were obtained from animals living in close contact with humans or from food: 7/10 pets, 4/6 zoo animals, 2/3 food samples. The detection of *Salmonella* spp. strains with tetracycline resistance genes in dogs could be interesting because often these pets share the dwelling, and in general live in close contact with their owners. This occurrence is relevant in the case of bacterial infections with oral-fecal transmission, such as *Salmonella* spp. Moreover, strains with these characteristics were isolated from animals that lived in a zoo, a place frequented by people, especially children, where the possibility of AMR spreading could increase.

Some serovars of *S. enterica* harboring *tet* genes have been isolated from livestock; among these five strains were harbored antibiotic resistance genes: two strains of *S.* Derby, one of *S.* Typhimurium monophasic variant, one of *S.* Montevideo and one of *S.* Muenster. Among these strains, the most representative gene was *tet* (D) (3/5). The presence of these genes in strains from livestock is not surprising, especially as these animals come into close contact with farmers. Certainly, this fact should be better investigated both because a transmission from these animals to humans and vice versa is possible, and also because during grazing these animals can contribute with their feces to the spread of ARGs on very large areas.

Other strains of *Salmonella* spp. examined in this study came from terrestrial wildlife; concerning these only a strain of *S.* Derby harbored *tet* (G), a tetracycline efflux protein found in Gram-negative bacteria. The encoding gene is found in both chromosomal and plasmid DNA where it is frequently linked to other genes which encode proteins that can confer florphenicol/chloramphenicol, sulfamethoxazole, and chloramphenicol resistance [36].

The *tet*(A) has been shown to be the most common genetic component in tetracycline-resistant *Escherichia coli* and *Salmonella* spp. [37]. Generally remaining in mobile genetic components (integrons, transposons, and plasmids), *tet* (A) can be easily transferred to different bacteria [38]. The ability of the *tet* (A) gene to spread freely in farm animals compared with other *tet* genes has been widely demonstrated [39]. Our results seem to confirm that this gene can be spread more easily in the environment.

Our data are in accordance with other reports, describing that tetracycline resistance is related to plasmid incompatibility; indeed, we never found strains containing simultaneously *tet* (A) and *tet* (B) genes either *tet* (C) and *tet* (D) [40]. Moreover, our data seem to confirm that *tet* (E) would not spread as easily as other *tet* genes since it is usually located on the chromosome on large, non-conjugative plasmids [41].

The presence of tetracycline resistance genes is alarming, since *Salmonella* can persist for months in adverse conditions and in the environment (i.e., non-drinking water), and thus can become responsible for intestinal infectious diseases in humans that often are considered as healthy carriers [3,28,29].

In addition to their high pathogenic potential, bacteria of the *Salmonella* genus are of particular interest for their contribution in the spread of antibiotic resistance, as they are able to accumulate and spread ARGs [42]. The genetic plasticity of *Salmonella* bacteria allows them to accumulate and disseminate ARGs that often are located in plasmids that carry also other virulence genes [43,44]. Thanks to this characteristic, the genus *Salmonella* spp. can easily transfer resistance genes horizontally to other bacteria [45]. Furthermore, the interaction between class 1 integrons and plasmids contributes to the ability of these bacteria to be reservoirs of ARGs [20,21]. For these reasons, the detection of *int1* was also conducted on all isolated strains. Our results showed that the strains isolated from wildlife did not harbor the *int1* gene, confirming the low presence of this genetic element in other bacterial strains collected from wild animals in Sicily [46]. The presence of *int1* was identified in 17/60 strains, in 8 of these it was in association with one or more *tet* genes, indicating a warning for the spread of ARGs, as noted elsewhere [47]. The frequency of class I integrons has been postulated as an indicator of anthropogenic pollution in the environment [48]. Indeed, the widespread presence of the *int1* gene in our samples highlights the potential transfer of ARGs between bacterial strains and their spread could increase the risk to human health. Concerning the high level of attention to antimicrobial resistance and the ease with which ARGs spread, from a One Health perspective it is important to monitor the presence of these genes, especially in zoonotic bacteria. Our data show that the majority of strains harboring antibiotic resistance genes have been isolated from animals in close contact with humans and that these animals could contribute significantly to the spreading of ARGs between bacteria.

## 4. Materials and Methods

### 4.1. Isolation and Identification of Salmonella spp.

Isolation of *Salmonella* spp. was conducted using enrichment broths and selective media as suggested by the OIE manual [3]. Colonies that could be attributed to *Salmonella* were identified at the genus level by biochemical testing (API20E^®^, bioMérieux, Craponne, France). Subsequently, the serotype of the *Salmonella* strains was determined using somatic O and flagellar H antisera (Becton Dickinson, Milano, Italy) [49]. All the media were purchased from Oxoid (Milano, Italy).

### 4.2. Antimicrobial Susceptibility by the Disk Diffusion Method

Antimicrobial susceptibility testing of 60 *Salmonella* isolates was performed on Mueller-Hinton agar (Oxoid, Milano, Italy) by disc diffusion method (Kirby-Bauer), according to Clinical and Laboratory Standard Institute (CLSI) guidelines [50]. A set of 16 antibiotics has been defined: sulfamethoxazole/trimethoprim (STX, 25 µg), kanamycin (K, 30 µg), gentamicin (CN, 10 µg), nalidixic acid (NA, 30 µg), tetracycline (TE, 30 µg), ampicillin (AMP, 10 µg), chloramphenicol (C, 10 µg), ciprofloxacin (CIP, 5 µg), cefotaxime (CTX, 30 µg), amoxicillin/clavulanic acid (AMC, 30 µg), enrofloxacin (ENR, 5 µg), tobramycin (TOB, 10 µg), levofloxacin (LEV, 5 µg), imipenem (IPM, 10 µg) and ceftriaxone (CRO, 30 µg) disks (Oxoid, Milano, Italy) were used.

A 0.5 McFarland bacterial suspension was prepared from each isolate in saline solution and spread on Mueller-Hinton agar. After incubation at 37 °C for 24 h, the isolates were classified as resistant (R), susceptible (S) or intermediate (I) after determination of the diameters of inhibition zones. The interpretation of the results was performed following CLSI indications [50].

### 4.3. DNA Extraction

Bacterial isolates were grown in Agar Nutrient (Oxoid, Milano, Italy) overnight. Two colonies from each plate were suspended in 100 µl of PrepMan™ Ultra Sample Preparation Reagent (Thermo Fisher Scientific, Waltham, MA, USA) following manufacturer’s instructions. The DNA obtained was stored at −20 °C until analysis.

### 4.4. Detection of Antibiotic Resistance Genes and Class-1 Integron

PCR reactions were carried out using 10 ng of DNA template and 0.5 μM of forward and reverse primers listed in Table 4, in a total volume of 25 µL of 1× of Advanced Universal SYBR Green Supermix (Bio-Rad Laboratories, Hercules, CA, USA), to amplify eight antibiotic resistance genes and the mobile element int1. The amplification of the 16S rDNA was used as a positive control. All samples considered positive to Real-Time PCR were verified by electrophoresis analysis on E-Gel™ Go! Agarose Gels, 2% (Thermo Fisher Scientific, Waltham, MA, USA).

To confirm the identity of the amplicons from tetracycline-susceptible strains, one amplicon of each *tet* gene was sequenced. DNA sequences were determined using the dideoxy chain termination method with a commercial DNA sequencing kit (BigDye™ Terminator v3.1 Cycle Sequencing Kit, Applied Biosystems™ (Thermo Fisher Scientific, Waltham, MA, USA) according to the manufacturer’s instructions. The obtained sequences were analyzed for nucleotide sequence identity by comparing them with reference strains in the GenBank database using the Basic Local Alignment Search Tool (BLAST) and The Comprehensive Antibiotic Resistance Database (The Comprehensive Antibiotic Resistance Database (https://card.mcmaster.ca, accessed on date 2 October 2021). Finally, the sequenced samples were used as positive controls to confirm by real-time PCR the presence of *tet* genes in tetracycline-susceptible strains. For this purpose, a sample was considered positive if the number of threshold cycles (Ct) was less than 35 cycles and if the melting curve had the same temperature and was overlapping with that one of the positive control (Supplementary Table S2).

## 5. Conclusions

The presence of *Salmonella* spp. in pets, or in animals living in close contact with humans, like livestock and zoo animals, suggests that the spread of these bacteria in animals should be better investigated. In fact, although infections caused by non-typhoidal *Salmonella* can have a paucisymptomatic or asymptomatic course in humans and do not cause disease in most animals, these bacteria would play a very important role in the environmental dissemination of tetracycline antibiotic resistance genes.

Therefore, the study of bacteria belonging to the genus *Salmonella* should always be done through a One Health approach, which considers the health of people, animals and the environment closely related, as these bacteria can be considered “sentinel” microorganisms for the spread of ARGs.

## Figures and Tables

**Table 1:** Source and serotypes of *Salmonella* spp. strains analyzed.

Origin	Serotypes Analyzed	Number of Strains
Livestock Animals (*n* = 16)	*S.* Derby	6
	*S.* Typhimurium Monophasic Variant	3
	*S*. Muenster	3
	*S.* Typhimurium	1
	*S.* Elomrane	1
	*S.* Montevideo	1
	*S.* Kedougou	1
Pets (*n* = 12)	*S.* Typhimurium	6
	*S.* Goldcoast	2
	*S.* Typhimurium Monophasic Variant	1
	*S.* Enteritidis	1
	*S.* Elomrane	1
	*S.* Schleissheim	1
Zoo Animals (*n* = 8)	*S.* Typhimurium	2
	*S.* Richmond	2
	*S*. Potsdam	1
	*S.* Kiambu	1
	*S*. Infantis	1
	*S.* Bahrenfeld	1
Wild Animals (*n* = 8)	*S.* Newport	1
	*S.* Heron	1
	*S. bongori*	1
	*S.* Elomrane	1
	*S.* Veneziana	1
	*S.* Abony	1
	*S.* Tennessee	1
	*S.* Enteritidis	1
Food from Animal Origin (*n* = 16)	*S.* Infantis	7
	*S.* Bredeney	2
	*S.* Kentucky	2
	*S.* Typhimurium	1
	*S.* Derby	1
	*S.* Newport	1
	*S.* Enteritidis	1
	*S.* Cardoner	1

**Table 2:** Phenotypic resistance results.

Id. Strain	Serotype	Phenotypic Resistance
Livestock Animals (*n* = 3/16)		
S1	*S.* Typhimurium Monophasic Variant	AMP
S2	*S.* Typhimurium Monophasic Variant	AMP
S64	*S.* Typhimurium Monophasic Variant	TE
Pets (*n* = 6/12)		
S3	*S.* Typhimurium	CN, TOB, AMP, TE
S19	*S.* Typhimurium	TE
S20	*S.* Typhimurium	TE
S24	*S.* Typhimurium	AMP, TE
S25	*S.* Typhimurium	AMP, TE
S32	*S.* Typhimurium	TE
Zoo Animals (*n* = 3/8)		
S26	*S.* Typhimurium	AMP, TE
S27	*S.* Typhimurium	AMP, TE
S43	*S*. Infantis	K, AMP, NA, SXT, TE
Wild Animals (*n* = 2/8)		
S4	*S.* Newport	TE
S41	*S.* Tennessee	NA
Food from Animal Origin (*n* = 9/16)		
S58	*S.* Newport	K, AMP, SXT, TE,
S62	*S.* Infantis	K, AMP, CTX, NA, TE
S63	*S.* Infantis	K, AMP, CTX, NA, SXT, TE
S48	*S.* Infantis	AMP, CTX, NA, SXT, TE
S55	*S.* Infantis	K, NA, SXT, TE
S56	*S.* Infantis	TE
S68	*S.* Infantis	NA, SXT, TE
S69	*S*. Infantis	K, NA, SXT, TE
S47	*S.* Derby	AMP, TE

Kanamycin (K); Gentamicin (CN); Tobramycin (TOB); Ampicillin (AMP); Cefotaxime (CTX); Nalidixic Acid (NA); Sulphamethoxazole/Trimethoprim (STX); Tetracycline (TE).

**Table 3:** Genotypic and phenotypic resistance to tetracycline results.

Id.	Source	Serovar	Tetracycline Resistance Genes	*int1*	Tetracycline Phenotypic Resistance
S1	Livestock	*S*. Typhimurium monofasic variant	*tet* (A)	*int1*	S
S7		*S*. Montevideo	*tet* (D)	*-*	S
S11		*S*. Derby	*tet* (G)	*int1*	S
S17		*S*. Muenster	*tet* (D)	*-*	S
S54		*S*. Derby	*tet* (E)*, tet* (D)	*-*	S
S3	Pets	*S*. Typhimurium monofasic variant	*tet* (B)*, tet* (G)	*-*	R
S8		*S*. Goldcoast	*tet* (A), *tet* (D)	*int1*	S
S9		*S*. Goldcoast	*tet* (A)	*int1*	S
S19		*S*. Typhimurium	*tet* (A), *tet* (E), *tet* (G), *tet* (C)	-	R
S20		*S*. Typhimurium	*tet* (E), *tet* (B), *tet* (C)	-	R
S24		*S*. Typhimurium	*tet* (A)	-	R
S25		*S*. Typhimurium	*tet* (A), *tet* (G)	-	R
S30		*S*. Enteritidis	*tet* (A), *tet* (E)	*int1*	S
S32		*S*. Typhimurium	*tet* (A)	-	R
S35		*S*. Elomrane	*tet* (C)	-	S
S15	Zoo	*S*. Kiambu	*tet* (G)	-	S
S26		*S*. Typhimurium	*tet* (A)	-	R
S27		*S*. Typhimurium	*tet* (A)	-	R
S29		*S*. Richmond	*tet* (A)	-	S
S43		*S*. Infantis	*tet* (A)	*int1*	R
S21		*S*. bongori	*tet* (C)	-	S
S47	Wildlife	*S*. Derby	*tet* (G)		R
S59	Food	*S*. Cardoner	*tet* (B)	-	S
S67		*S*. Enteritidis	*tet* (A)	*int1*	S
S69		*S*. Infantis	*tet* (A)	*int1*	R

Resistant (R); Susceptible (S).

**Table 4:** Primers and the annealing temperatures used in this study.

Target Gene	Primer Sequence (5′–3′)	Function	Annealing Temperature (°C)	Amplicon Size (bp)	References
*tet* (A)	GCTACATCCTGCTTGCCTTC CATAGATCGCCGTGAAGAGG	Efflux	60	210	[51]
*tet* (B)	TTGGTTAGGGGCAAGTTTTG GTAATGGGCCAATAACACCG	Efflux	60	659	[51]
*tet* (C)	CTTGAGAGCCTTCAACCCAG ATGGTCGTCATCTACTGCC	Efflux	60	418	[51]
*tet* (D)	AAACCATTACGGCATTCTGC GACCGGATACACCATCCATC	Efflux	60	787	[52]
*tet* (E)	AAACCACATCCTCCATACGC AAATAGGCCACAACCGTCAG	Efflux	60	278	[52]
*tet* (G)	GCTCGGTGGTATCTCTGCTC AGCAACAGAATCGGGAACAC	Efflux	60	844	[52]
*tet* (O)	GGAGGGGTTCAACCACAAAG CTATGTAAATAAAATGGATAG	Ribosomal protection	55	88	[53]
*tet* (W)	ACATCATTGATACTCCAGGTCACG TTTCACTTTGTGGTTGAACCCCTC	Ribosomal protection	60	142	[53]
*int1*	CCT CCC GCA CGA TGA TC TCC ACG CAT CGT CAG GC	Class 1 integron	60	280	[53]
*16S rDNA*	CGGTGAATACGTTCYCGG GGHTACCTTGTTACGACTT	Positive Control	*55*	142	[47]

## Data Availability

All data discussed are contained in the article.

## Supplementary Materials

The following are available online at https://www.mdpi.com/article/10.3390/antibiotics10070809/s1, Table S1: Results of antimicrobial susceptibility by the Kirby-Bauer method; Table S2: Real-time PCR data for *tet* (A) and *tet* (B) genes in tetracycline-susceptible strains.
