# Supplementary material for: Antibiotic Susceptibility Profile and Tetracycline Resistance Genes Detection in Salmonella spp. Strains Isolated from Animals and Food
Source: Antibiotics (Basel). 2021 Jul 2;10(7):809. doi: 10.3390/antibiotics10070809 (PMC8300615; doi:10.3390/antibiotics10070809)
Supplement: Supplementary file 1 [file antibiotics-10-00809-s001.zip › Supplementary Material Table S1.pdf]

Supplementary Material - Table S1. Results of antimicrobial susceptibility by the Kirby-Bauer method.

Id. strain	Animal	Serotyping	K	CN	TOB	AMP	AMC	CTX	CRO	IMP	NA	CIP	ENR	LEV	SXT	TE	C
**Livestock animals (n=16)**																	
**S1**	Ovine	*S.* Typhimurium Monophasic Variant	I	S	S	**R**	S	S	S	S	S	S	S	S	S	S	S
**S2**	Ovine	*S.* Typhimurium Monophasic Variant	I	S	S	**R**	S	S	S	S	S	S	S	S	S	S	S
**S7**	Ovine	*S.* Montevideo	S	S	S	S	S	S	S	S	S	S	S	S	S	I	S
**S11**	Ovine	*S.* Derby	S	S	S	S	S	S	S	S	S	S	S	S	S	S	S
**S12**	Ovine	*S.* Elomrane	S	S	S	S	S	S	S	S	S	S	S	S	S	S	S
**S16**	Ovine	*S.* Muenster	S	S	S	S	S	S	S	S	S	S	S	S	S	S	I
**S17**	Ovine	*S.* Muenster	S	S	S	S	S	S	S	S	S	S	S	S	S	S	S
**S40**	Ovine	*S.* Derby	S	S	S	S	S	S	S	S	S	S	S	S	S	S	S
**S38**	Bovine	*S.* Muenster	S	S	S	S	S	S	S	S	S	S	S	S	S	S	S
**S49**	Swine	*S.* Derby	S	S	S	S	S	S	S	S	S	S	S	S	S	S	S
**S50**	Swine	*S.* Derby	S	S	S	S	S	S	S	S	S	S	S	S	S	S	S
**S51**	Swine	*S.* Typhimurium	I	S	S	S	S	S	S	S	S	S	S	S	S	S	S
**S54**	Swine	*S.* Derby	S	S	S	S	S	S	S	S	S	S	S	S	S	S	S
**S60**	Swine	*S.* Derby	I	S	S	S	S	S	S	S	S	S	S	S	S	S	S
**S64**	Swine	*S.* Typhimurium Monophasic Variant	S	S	S	S	S	S	S	S	S	S	S	S	S	**R**	S
**S70**	Swine	*S.* Kedougou	I	S	S	S	S	S	S	S	S	S	S	S	S	S	S
**Pets (n=12)**																	
**S3**	Dog	*S.* Typhimurium Monophasic Variant	I	**R**	**R**	**R**	S	S	S	S	S	S	S	S	S	**R**	I
**S8**	Dog	*S.* Goldcoast	S	S	S	S	S	S	S	S	S	S	S	S	S	S	S
**S9**	Dog	*S.* Goldcoast	S	S	S	S	S	S	S	S	S	S	S	S	S	S	S
**S19**	Dog	*S.* Typhimurium	S	S	S	S	S	S	S	S	S	S	S	S	S	**R**	S
**S20**	Dog	*S.* Typhimurium	S	S	S	S	S	S	S	S	S	S	S	S	S	**R**	S
**S24**	Dog	*S.* Typhimurium	S	S	S	**R**	S	S	S	S	S	S	S	S	S	**R**	S
**S25**	Dog	*S.* Typhimurium	S	S	S	**R**	S	S	S	S	S	S	S	S	S	**R**	S
**S30**	Dog	*S.* Enteritidis	S	S	S	S	S	S	S	S	S	S	S	S	S	S	S
**S32**	Dog	*S.* Typhimurium	S	S	S	S	S	S	S	S	S	S	S	S	S	**R**	S

**S34**	Dog	*S.* Typhimurium	S	S	S	S	S	S	S	S	S	S	S	S	S	S	S
**S35**	Dog	*S.* Elomrane	S	S	S	S	S	S	S	S	S	S	S	S	S	S	S
**S42**	Dog	*S.* Schleissheim	S	S	S	S	S	S	S	S	S	S	S	S	S	S	S
**Zoo Animals (n=8)**																	
**S6**	*Python regius*	*S.* Bahrenfeld	S	S	S	S	S	S	S	S	S	S	S	S	S	S	S
**S13**	*Trachemys scripta*	*S.* Richmond	S	S	S	S	S	S	S	S	S	S	S	S	S	S	S
**S14**	*Iguana iguana*	*S.* Potsdam	S	S	S	S	S	S	S	S	S	S	S	S	S	S	S
**S15**	*Trachemys scripta*	*S.* Kiambu	S	S	S	S	S	S	S	S	S	S	S	S	S	S	S
**S29**	*Trachemys scripta*	*S.* Typhimurium	S	S	S	**R**	S	S	S	S	S	S	S	S	S	**R**	S
**S26**	*Carduelis carduelis*	*S.* Typhimurium	S	S	S	**R**	S	S	S	S	S	S	S	S	S	**R**	S
**S27**	*Nymphicus hollandicus*	*S.* Richmond	S	S	S	S	S	S	S	S	S	S	S	S	S	S	S
**S43**	*Carduelis carduelis*	*S.* Infantis	**R**	S	S	**R**	S	S	S	S	**R**	S	S	S	**R**	**R**	S
**Wild animals (n=8)**																	
**S4**	*Testudo hermanni*	*S.* Newport	S	S	S	S	S	S	S	S	S	S	S	S	S	**R**	S
**S5**	*Testudo hermanni*	*S.* Heron	S	S	S	S	S	S	S	S	S	S	S	S	S	S	S
**S21**	*Testudo hermanni*	*S. bongori*	S	S	S	S	S	S	S	S	S	S	S	S	S	S	S
**S31**	*Sus scrofa*	*S.* Elomrane	S	S	S	S	S	S	S	S	S	S	S	S	S	S	S
**S36**	*Oryctolagus cuniculus*	*S.* Veneziana	S	S	S	S	S	S	S	S	S	S	S	S	S	S	S
**S39**	*Testudo hermanni*	*S.* Abony	S	S	S	S	S	S	S	S	S	S	S	S	S	S	S
**S41**	*Testudo hermanni*	*S.* Tennessee	I	S	S	I	S	S	S	S	**R**	S	S	S	S	I	S
**S44**	*Ardea cinerea*	*S.* Enteritidis	S	S	S	S	S	S	S	S	S	S	S	S	S	S	S
**Food from animal origin (n=16)**																	
**S46**	Turkey meat	*S.* Typhimurium	I	S	S	S	S	S	S	S	S	S	S	S	S	S	S
**S58**	Turkey meat	*S.* Newport	**R**	S	S	**R**	S	S	S	S	I	S	S	S	**R**	**R**	S
**S62**	Bovine meat	*S.* Infantis	**R**	S	S	**R**	I	**R**	S	S	**R**	S	S	S	S	**R**	S
**S63**	Bovine meat	*S.* Infantis	**R**	S	S	**R**	I	**R**	S	S	**R**	S	S	S	**R**	**R**	S
**S48**	Chicken meat	*S.* Infantis	I	S	S	**R**	S	**R**	S	S	**R**	S	S	S	**R**	**R**	S
**S55**	Chicken meat	*S.* Infantis	**R**	S	S	S	S	S	S	S	**R**	S	S	S	**R**	**R**	S
**S56**	Chicken meat	*S.* Infantis	I	S	S	S	S	S	S	S	S	S	S	S	S	**R**	S

**S65**	Chicken meat	*S.* Kentucky	I	S	S	S	S	S	S	S	S	S	S	S	S	S	S
**S66**	Chicken meat	*S.* Kentucky	I	S	S	S	S	S	S	S	S	S	S	S	S	S	S
**S68**	Chicken meat	*S.* Infantis	S	S	S	S	S	S	S	S	**R**	S	S	S	**R**	**R**	S
**S69**	Chicken meat	*S.* Infantis	**R**	S	S	S	S	S	S	S	**R**	S	S	S	**R**	**R**	S
**S67**	Eggs	*S.* Eenteritidis	S	S	S	S	S	S	S	S	S	S	S	S	S	S	S
**S47**	Mytiles	*S.* Derby	I	S	S	**R**	S	S	S	S	S	S	S	S	S	**R**	S
**S52**	Mytiles	*S.* Bredeney	I	S	S	S	S	S	S	S	S	S	S	S	S	S	S
**S53**	Mytiles	*S.* Bredeney	S	S	S	S	S	S	S	S	S	S	S	S	S	S	S
**S59**	Clams	*S.* Cardoner	S	S	S	S	S	S	S	S	S	S	S	S	S	S	S

Kanamycin (K); Gentamicin (CN); Tobramycin (TOB); Ampicillin (AMP); Amoxicillin + clavulanic acid (AMC); Cefotaxime (CTX); Ceftriaxone (CRO); Imipenem (IMP); Nalidixic acid (NA); Ciprofloxacin (CIP); Enrofloxacin (ENR); Levofloxacin (LEV); Sulfamethoxazole + Thrimethoprim (SXT); Tetracycline (TE); Chloramphenicol (C); Resistant (R); Susceptible (S); Intermediate (I).
