# Supplementary material for: Antibiotic Susceptibility Profile and Tetracycline Resistance Genes Detection in Salmonella spp. Strains Isolated from Animals and Food
Source: Antibiotics (Basel). 2021 Jul 2;10(7):809. doi: 10.3390/antibiotics10070809 (PMC8300615; doi:10.3390/antibiotics10070809)
Supplement: Supplementary file 1 [file antibiotics-10-00809-s001.zip › Supplementary Material Table S2.pdf]

Supplementary Material - Table S2. Real-time PCR data for *tet*(A) and *tet*(B) genes in tetracycline-susceptible strains

Salmonella ID	Gene	Ct	Melting Temperature (°C)
S1_Positive Control Sequenced	tet(A)	28.79	87.50 – 88.50
S8	tet(A)	30.98	
S9	tet(A)	28.96	
S30	tet(A)	31.00	
S29	tet(A)	29.19	
S67	tet(A)	22.73	
S59_Positive Control sequenced	tet(B)	26.99	82.50 -83.00
-	NTC_tet(A)	N/A	--
-	NTC_tet(B)	N/A	--
